# Herpes and Chest Pain: Two Atypical Monkeypox Cases

**DOI:** 10.7759/cureus.33705

**Published:** 2023-01-12

**Authors:** Devin Videlefsky, Constangela Matos Noboa, Charu Ramchandani

**Affiliations:** 1 Department of Medicine, Albert Einstein College of Medicine, Bronx, USA; 2 Department of Medicine, Montefiore Medical Center, Wakefield Campus, Bronx, USA

**Keywords:** mpox, genital lesion, cardiac troponin, herpes simplex virus infection, hiv, monkeypox

## Abstract

The 2022 Monkeypox Outbreak has spread globally in just a few months and has raised great concerns regarding disease recognition due to frequent atypical presentations and questions regarding the possibility of sexual transmission. In endemic countries and prior outbreaks, the clinical manifestations of monkeypox have been well documented, with cutaneous findings following a set, synchronous pattern of evolution. We present two cases of atypical monkeypox presentations in individuals living with HIV, both complicated by herpes simplex virus type 2 (HSV-2) coinfection and elevated troponins, and both demonstrating the ease with which monkeypox can be overlooked in the current outbreak.

## Introduction

The 2022 Monkeypox Outbreak was first confirmed in May 2022 in the United Kingdom [1]. In the months following, the outbreak spread to various non-endemic countries, with more than 80,000 cases reported worldwide, and has been declared a public health emergency by the World Health Organization [2,3]. Monkeypox is a viral disease caused by the monkeypox virus. The typical monkeypox infection consists of an incubation period of five to 13 days after the exposure, followed by a prodrome of flu-like symptoms and lymphadenopathy lasting one to four days. This is followed by two to three weeks of systemic disease characterized by the distinctive monkeypox rash appearing one to three days following the development of fever. Historically, the classic rash most often affects the face (95%), palms of hands (75%), and oral mucosa rather than the trunk and genitalia. The lesions typically follow a synchronized evolution pattern through four stages: macular, papular, vesicular, to pustular, before ultimately scabbing over and desquamating [4]. The 2022 Monkeypox Outbreak has been notable for atypical presentations that have differed widely from the classic disease presentation and course. In this report, we present two cases of male individuals, both with HIV and in a monogamous relationship, who both had atypical presentations of monkeypox infection, each complicated by herpes simplex virus type 2 (HSV-2) coinfection and elevated cardiac enzymes.

This article was previously presented as a poster abstract at the 2022 NYACP Annual Scientific Meeting on November 5, 2022.

## Case presentation

Case one

Patient A was a middle-aged male with a past medical history of HIV [on antiretroviral therapy (ART) with a CD4 count of 1,171 cells/uL], asthma, and obstructive sleep apnea. Of note, the patient had undergone a cardiac catheterization procedure one year prior for anginal-like chest pain, which had shown no obstruction or intraluminal irregularities of the coronary arteries, with symptoms having completely resolved since. The patient presented with five days of sore throat, fever, myalgias, headache, and intermittent pleuritic chest pain. In the emergency department, the patient endorsed progressively worsening truncal rash, groin pain and edema, and penile discharge for three days. He stated that the skin lesions had also spread to his chest. On arrival, the patient was febrile, with otherwise normal vital signs. Physical exam was notable for an erythematous, non-pruritic nodular rash on the chest, back, trunk, and extremities with small discrete, non-tender, skin-colored lesions over the affected area, some with umbilication. The patient lacked oral or pharyngeal lesions. The genital exam showed a painless skin-colored papule on tip of the glans penis, a painful erythematous ulcer in the dorsal aspect glans penis, whitish penile discharge, and bilateral painful inguinal lymphadenopathy. Initial laboratory tests were notable for an elevated troponin I of 0.38 ng/mL (reference range: <0.03 ng/mL). Other relevant laboratory tests were at the patient's baseline and within normal limits, including a creatinine level of 0.95 mg/dL and a brain natriuretic peptide level of 79 pg/mL. The patient had no prior troponin levels available to assess the baseline. The patient was admitted to the telemetry unit to rule out acute cardiac etiology for his chest pain and troponinemia.

Case two

Patient B was a young adult male with a past medical history of HIV on ART (diagnosed three months prior with a recent CD4 count of 667 cells/uL), previously treated syphilis infection, obesity, obstructive sleep apnea, and history of external hemorrhoids. He had no prior surgical history and stated that he is in a monogamous relationship with Patient A with inconsistent use of barrier protection. He had also recently attended several crowded social gatherings with a high risk of skin-to-skin contact with other attendees. He presented with similar flu-like symptoms for four days and constant, non-radiating, dull, left-sided groin pain and rectal pain for two days. On arrival at the hospital, the patient was febrile and endorsed a sudden and brief episode of sharp chest pain lasting a few seconds that resolved spontaneously. A physical exam showed tender lymphadenopathy in the right groin and mild erythematous irritation of the perianal skin. There were no other skin or anogenital lesions or discharge present. He also had elevated troponin I of 0.22 ng/mL, which rose to 0.51 ng/mL a few hours later. Other relevant laboratory tests, including creatinine, were at baseline and within normal limits. The patient was admitted to the telemetry unit. On day two of hospitalization, he developed two small erythematous papules on his right forearm and foot.

Hospital course, test results, and treatment

Following a negative cardiac workup, including unremarkable echocardiograms and electrocardiograms (ECGs), and down-trending troponins for both patients, the elevated troponins were likely due to demand ischemia in the setting of infection or very less likely secondary to mild myocarditis. No further cardiac workup was done given the lack of arrhythmias, ECG changes, or signs of cardiac dysfunction. Multiple swabs were taken from the penile, rectal, and skin lesions and were sent for monkeypox and common sexually transmitted infections (STI) testing. A repeat monkeypox swab was also obtained 12 hours after the first as per protocol. Both patients tested positive for HSV-2 and were treated with the standard therapy of valacyclovir. With this test result, and improvement in symptoms, monkeypox was considered unlikely and both patients were discharged home with instructions to quarantine as a precaution. Following a two-week turnaround, monkeypox PCR tests for both patients returned positive for all samples, including the repeat swab. Follow-up communication revealed the resolution of symptoms, with skin lesions having scabbed over and desquamated.

## Discussion

During the 2022 Monkeypox Outbreak, cases have proven to be difficult to identify and diagnose. The presentation of both patients discussed here not only differed from the classic monkeypox presentation but also differed from each other. Patient A and Patient B both experienced the classic prodrome of flu-like symptoms; however, only Patient A had early and widespread cutaneous findings. Patient B only developed two skin lesions, more than one week after developing a fever. As mentioned before, the rash tends to emerge one to three days after the first fever spike and usually affects the face, oral mucosa, and extremities [3]. Patient A developed skin lesions on his genital area and trunk that only subsequently spread to his chest and proximal extremities. The skin lesions in either patient failed to follow the synchronized evolution pattern associated with monkeypox. The lesions appeared at all different stages, with many skin lesions skipping various stages altogether. This atypical presentation has been noted in some recent reports [5]. Clinicians who are unaware of the emerging atypical rashes may fail to consider monkeypox when encountering a rash that does not follow the typical pattern.

When questioning the validity of the monkeypox PCR test results, many factors were considered. Ultimately, given that multiple swabs were positive for monkeypox, including the repeat swab, as well as the higher monkeypox prevalence at this time during the height of the outbreak, and high-risk patient behaviors, the test results were considered most likely to be true-positive results.

One aspect of Patient B’s presentation that is atypical for monkeypox was his immense rectal pain following fever onset, which was his major complaint on admission. Although this patient did have a history of external hemorrhoids, he stated that this pain felt different from his usual symptoms. The 2022 Outbreak has been noted to disproportionately affect persons who identify as gay, bisexual, or as men who have sex with men (MSM), with a majority of cases suspected to be transmitted through sexual activity [6]. One review of 124 patients found sexual exposure documented in 91.67% of cases [7]. Both our patients had been sexually active with each other in the weeks prior to admission and endorsed inconsistent use of barrier protection. While both patients reported being monogamous, they may have contracted the virus through non-sexual skin-to-skin contact at social gatherings. If this is the case, it is extremely interesting that the virus manifested with anogenital symptoms. Patient B’s report of severe anorectal pain has been similar to other observational reports published during the 2022 Outbreak, one even postulating that the majority of admissions were due to rectal pain management [8].

While much is still unknown about the modes of monkeypox transmission during the 2022 Outbreak, more investigations are needed to determine the connection between the overwhelming anogenital symptoms and the high incidence of sexual exposure among patients. One study found that of 32 monkeypox patients who had semen samples tested, 29 tested positive for monkeypox viral DNA [9]. To date, there is no evidence available for the infectiousness of semen; however, it is clear that sexual activity is a huge factor, and further investigation into the possibility of classifying monkeypox as an STI is needed. In patients who are at high risk for monkeypox infection and who complain of fever and rectal pain without skin lesions, monkeypox should be on the differential, as these lesions may not have emerged yet, or maybe hidden within the rectal mucosa.

Another feature of a possible association with sexual activity that was revealed in these two cases was the concomitant infection with HSV-2. Although monkeypox suspicion was high initially, it gradually diminished due to the peculiar presentations and positive HSV-2 test result that could explain the majority of the patient’s symptoms. Primary HSV-2 infection can present with fever, malaise, headache, myalgias, painful genital ulcers, tender inguinal lymphadenopathy, and commonly cause proctitis and rectal pain in MSM, all of which were seen here. Patient A’s painful erythematous ulcer on the dorsal aspect of the glans penis was very similar to typical genital herpes ulcers. While our ultimate diagnosis of genital herpes was not incorrect, it led to a premature exclusion of a concomitant monkeypox infection, which could also cause similar symptoms. In fact, one cohort study of 528 monkeypox patients reported that 73% of patients had anogenital lesions with 54 patients having a single genital ulcer as their only cutaneous sign. The same study found that 29% of patients were also diagnosed with other STIs at the time of their monkeypox diagnosis [9]. This data shows how easy it is to not only misdiagnose monkeypox as another common STI but to also exclude monkeypox in the setting of a proven STI, when the patient may be coinfected with both pathogens.

Both patients had elevated cardiac biomarkers and complained of atypical chest pain, initially raising concern for a possible acute coronary syndrome (ACS) or myocarditis. Suspicion for ACS was very low from the beginning; however, the presence of elevated troponins did indicate some form of cardiac injury, requiring a workup. Cardiac manifestations of monkeypox have only been described in a handful of cases, all relating to possible myocarditis [10,11]. While it is true that HSV-2 may also cause myocarditis, the patients never showed vital sign abnormalities, ECG, or echocardiogram consistent with such a diagnosis. While the troponinemia, in this case, was most likely in the setting of demand ischemia, the atypical chest pain remained somewhat of a mystery. Further investigation is needed to determine whether there is a component of the monkeypox virus causing direct cardiac tissue injury or if the elevated troponins were due to demand ischemia in the setting of infection.

Although these patients had good immunological status with regard to their HIV and high CD4 cell count, minimal information is available relating to the extent to which HIV status changes the risk of acquiring monkeypox or one’s risk of developing severe systemic disease [12]. The aforementioned study of 528 patients found that patients diagnosed with HIV accounted for 41% of cases. They also found no difference between clinical features of monkeypox in patients with HIV and those without; however, 99% of those studied were on ART and 89% had CD4 counts >500 cells/uL. Of note, one HIV-positive patient in the study did develop myocarditis, with a CD4 count of 780 cells/uL [9]. As the number of monkeypox cases increases, further review of the disease course among patients diagnosed with HIV may enable the identification of patients at high risk of serious complications.

## Conclusions

These two cases illustrate many important caveats of possible presentations in the setting of the 2022 Monkeypox Outbreak. Firstly, while both patients had the classic viral prodrome of monkeypox, their cutaneous manifestations did not follow the typical set evolution pattern and involved atypical areas of the body. Secondly, Patient B’s chief symptom was rectal pain, with minimal cutaneous manifestations. Thirdly, clinicians should be wary of disregarding a possible monkeypox infection in the presence of a more common STI, as these patients can have a concurrent monkeypox infection. Finally, a link between the monkeypox virus and elevated troponins has not been well documented and further investigation is needed to ascertain a possible connection between the two.
